# The impact of adverse childhood experiences on multimorbidity: a systematic review and meta-analysis

**DOI:** 10.1186/s12916-024-03505-w

**Published:** 2024-08-15

**Authors:** Dhaneesha N. S. Senaratne, Bhushan Thakkar, Blair H. Smith, Tim G. Hales, Louise Marryat, Lesley A. Colvin

**Affiliations:** 1 Chronic Pain Research Group, Division of Population Health & Genomics, School of Medicine, University of Dundee, Ninewells Hospital, Dundee, DD1 9SY UK; 2 Institute of Academic Anaesthesia, Division of Systems Medicine, School of Medicine, University of Dundee, Dundee, UK; 3 School of Health Sciences, University of Dundee, Dundee, UK

**Keywords:** Adverse childhood experiences, Childhood adversity, Chronic disease, Long-term conditions, Multimorbidity

## Abstract

**Background:**

Adverse childhood experiences (ACEs) have been implicated in the aetiology of a range of health outcomes, including multimorbidity. In this systematic review and meta-analysis, we aimed to identify, synthesise, and quantify the current evidence linking ACEs and multimorbidity.

**Methods:**

We searched seven databases from inception to 20 July 2023: APA PsycNET, CINAHL Plus, Cochrane CENTRAL, Embase, MEDLINE, Scopus, and Web of Science. We selected studies investigating adverse events occurring during childhood (< 18 years) and an assessment of multimorbidity in adulthood (≥ 18 years). Studies that only assessed adverse events in adulthood or health outcomes in children were excluded. Risk of bias was assessed using the ROBINS-E tool. Meta-analysis of prevalence and dose–response meta-analysis methods were used for quantitative data synthesis. This review was pre-registered with PROSPERO (CRD42023389528).

**Results:**

From 15,586 records, 25 studies were eligible for inclusion (total participants = 372,162). The prevalence of exposure to ≥ 1 ACEs was 48.1% (95% CI 33.4 to 63.1%). The prevalence of multimorbidity was 34.5% (95% CI 23.4 to 47.5%). Eight studies provided sufficient data for dose–response meta-analysis (total participants = 197,981). There was a significant dose-dependent relationship between ACE exposure and multimorbidity (*p* < 0.001), with every additional ACE exposure contributing to a 12.9% (95% CI 7.9 to 17.9%) increase in the odds for multimorbidity. However, there was heterogeneity among the included studies (*I*^2^ = 76.9%, Cochran *Q* = 102, *p* < 0.001).

**Conclusions:**

This is the first systematic review and meta-analysis to synthesise the literature on ACEs and multimorbidity, showing a dose-dependent relationship across a large number of participants. It consolidates and enhances an extensive body of literature that shows an association between ACEs and individual long-term health conditions, risky health behaviours, and other poor health outcomes.

**Supplementary Information:**

The online version contains supplementary material available at 10.1186/s12916-024-03505-w.

## Background

In recent years, adverse childhood experiences (ACEs) have been identified as factors of interest in the aetiology of many conditions [1]. ACEs are potentially stressful events or environments that occur before the age of 18. They have typically been considered in terms of abuse (e.g. physical, emotional, sexual), neglect (e.g. physical, emotional), and household dysfunction (e.g. parental separation, household member incarceration, household member mental illness) but could also include other forms of stress, such as bullying, famine, and war. ACEs are common: estimates suggest that 47% of the UK population have experienced at least one form, with 12% experiencing four or more [2]. ACEs are associated with poor outcomes in a range of physical health, mental health, and social parameters in adulthood, with greater ACE burden being associated with worse outcomes [1–8].

Over a similar timescale, multimorbidity has emerged as a significant heath challenge. It is commonly defined as the co-occurrence of two or more long-term conditions (LTCs), with a long-term condition defined as any physical or mental health condition lasting, or expected to last, longer than 1 year [9]. Multimorbidity is both common and age-dependent, with a global adult prevalence of 37% that rises to 51% in adults over 60 [10, 11]. Individuals living with multimorbidity face additional challenges in managing their health, such as multiple appointments, polypharmacy, and the lack of continuity of care [12–14]. Meanwhile, many healthcare systems struggle to manage the additional cost and complexity of people with multimorbidity as they have often evolved to address the single disease model [15, 16]. As global populations continue to age, with an estimated 2.1 billion adults over 60 by 2050, the pressures facing already strained healthcare systems will continue to grow [17]. Identifying factors early in the aetiology of multimorbidity may help to mitigate the consequences of this developing healthcare crisis.

Many mechanisms have been suggested for how ACEs might influence later life health outcomes, including the risk of developing individual LTCs. Collectively, they contribute to the idea of ‘toxic stress’; cumulative stress during key developmental phases may affect development [18]. ACEs are associated with measures of accelerated cellular ageing, including changes in DNA methylation and telomere length [19, 20]. ACEs may lead to alterations in stress-signalling pathways, including changes to the immune, endocrine, and cardiovascular systems [21–23]. ACEs are also associated with both structural and functional differences in the brain [24–27]. These diverse biological changes underpin psychological and behavioural changes, predisposing individuals to poorer self-esteem and risky health behaviours, which may in turn lead to increased risk of developing individual LTCs [1, 2, 28–32]. A growing body of evidence has therefore led to an increased focus on developing trauma-informed models of healthcare, in which the impact of negative life experiences is incorporated into the assessment and management of LTCs [33].

Given the contributory role of ACEs in the aetiology of individual LTCs, it is reasonable to suspect that ACEs may also be an important factor in the development of multimorbidity. Several studies have implicated ACEs in the aetiology of multimorbidity, across different cohorts and populations, but to date no meta-analyses have been performed to aggregate this evidence. In this review, we aim to summarise the state of the evidence linking ACEs and multimorbidity, to quantify the strength of any associations through meta-analysis, and to highlight the challenges of research in this area.

## Methods

### Search strategy and selection criteria

We conducted a systematic review and meta-analysis that was prospectively registered in the International Prospective Register of Systematic Reviews (PROSPERO) on 25 January 2023 (ID: CRD42023389528) and reported using the Preferred Reporting Items for Systematic Reviews and Meta-Analyses (PRISMA) guidelines.

We developed a search strategy based on previously published literature reviews and refined it following input from subject experts, an academic librarian, and patient and public partners (Additional File 1: Table S1). We searched the following seven databases from inception to 20 July 2023: APA PsycNET, CINAHL Plus, Cochrane CENTRAL, Embase, MEDLINE, Scopus, and Web of Science. The search results were imported into Covidence (Veritas Health Innovation, Melbourne, Australia), which automatically identified and removed duplicate entries. Two reviewers (DS and BT) independently performed title and abstract screening and full text review. Discrepancies were resolved by a third reviewer (LC).

Reports were eligible for review if they included adults (≥ 18 years), adverse events occurring during childhood (< 18 years), and an assessment of multimorbidity or health status based on LTCs. Reports that only assessed adverse events in adulthood or health outcomes in children were excluded.

The following study designs were eligible for review: randomised controlled trials, cohort studies, case–control studies, cross-sectional studies, and review articles with meta-analysis. Editorials, case reports, and conference abstracts were excluded. Systematic reviews without a meta-analysis and narrative synthesis review articles were also excluded; however, their reference lists were screened for relevant citations.

### Data analysis

Two reviewers (DS and BT) independently performed data extraction into Microsoft Excel (Microsoft Corporation, Redmond, USA) using a pre-agreed template. Discrepancies were resolved by consensus discussion with a third reviewer (LC). Data extracted from each report included study details (author, year, study design, sample cohort, sample size, sample country of origin), patient characteristics (age, sex), ACE information (definition, childhood cut-off age, ACE assessment tool, number of ACEs, list of ACEs, prevalence), multimorbidity information (definition, multimorbidity assessment tool, number of LTCs, list of LTCs, prevalence), and analysis parameters (effect size, model adjustments). For meta-analysis, we extracted ACE groups, number of ACE cases, number of multimorbidity cases, number of participants, odds ratios or regression beta coefficients, and 95% confidence intervals (95% CI). Where data were partially reported or missing, we contacted the study authors directly for further information.

Two reviewers (DS and BT) independently performed risk of bias assessments of each included study using the Risk Of Bias In Non-randomized Studies of Exposures (ROBINS-E) tool [34]. The ROBINS-E tool assesses the risk of bias for the study outcome relevant to the systematic review question, which may not be the primary study outcome. It assesses risk of bias across seven domains; confounding, measurement of the exposure, participant selection, post-exposure interventions, missing data, measurement of the outcome, and selection of the reported result. The overall risk of bias for each study was determined using the ROBINS-E algorithm. Discrepancies were resolved by consensus discussion.

All statistical analyses were performed in R version 4.2.2 using the RStudio integrated development environment (RStudio Team, Boston, USA). To avoid repetition of participant data, where multiple studies analysed the same patient cohort, we selected the study with the best reporting of raw data for meta-analysis and the largest sample size. Meta-analysis of prevalence was performed with the meta package [35], using logit transformations within a generalised linear mixed model, and reporting the random-effects model [36]. Inter-study heterogeneity was assessed and reported using the *I*^2^ statistic, Cochran *Q* statistic, and Cochran *Q p*-value. Dose–response meta-analysis was performed using the dosresmeta package [37] following the method outlined by Greenland and Longnecker (1992) [38, 39]. Log-linear and non-linear (restricted cubic spline, with knots at 5%, 35%, 65%, and 95%) random effects models were generated, and goodness of fit was evaluated using a Wald-type test (denoted by *X*^2^) and the Akaike information criterion (AIC) [39].

### Patient and public involvement

The Consortium Against Pain Inequality (CAPE) Chronic Pain Advisory Group (CPAG) consists of individuals with lived experiences of ACEs, chronic pain, and multimorbidity. CPAG was involved in developing the research question. The group has experience in systematic review co-production (in progress).

## Results

**Fig. 1:**
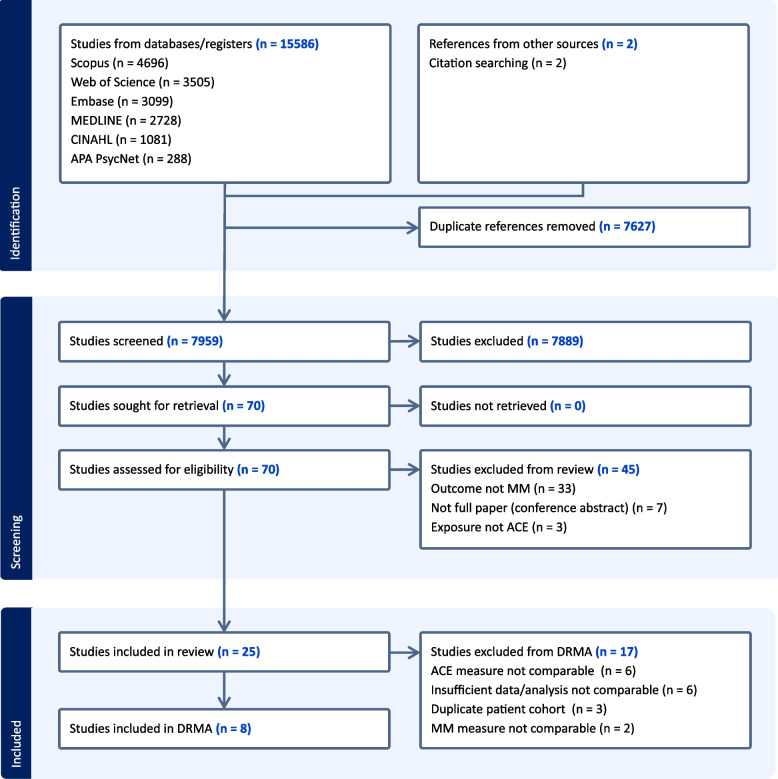
Flow chart of selection of studies into the systematic review and meta-analysis. Flow chart of selection of studies into the systematic review and meta-analysis. ACE, adverse childhood experience; MM, multimorbidity; DRMA, dose–response meta-analysis

The search identified 15,586 records, of which 25 met inclusion criteria for the systematic review (Fig. 1) [40–64]. The summary characteristics can be found in Additional File 1: Table S2. Most studies examined European (*n* = 11) or North American (*n* = 9) populations, with a few looking at Asian (*n* = 3) or South American (*n* = 1) populations and one study examining a mixed cohort (European and North American populations). The total participant count (excluding studies performed on the same cohort) was 372,162. Most studies had a female predominance (median 53.8%, interquartile range (IQR) 50.9 to 57.4%).

All studies were observational in design, and so risk of bias assessments were performed using the ROBINS-E tool (Additional File 1: Table S3) [34]. There were some consistent risks observed across the studies, especially in domain 1 (risk of bias due to confounding) and domain 3 (risk of bias due to participant selection). In domain 1, most studies were ‘high risk’ (*n* = 24) as they controlled for variables that could have been affected by ACE exposure (e.g. smoking status) [40, 41, 43–64]. In domain 3, some studies were ‘high risk’ (*n* = 7) as participant selection was based on participant characteristics that could have been influenced by ACE exposure (e.g. through recruitment at an outpatient clinic) [45, 48, 49, 51, 53, 54, 58]. The remaining studies were deemed as having ‘some concerns’ (*n* = 18) as participant selection occurred at a time after ACE exposure, introducing a risk of survivorship bias [40–44, 46, 47, 50, 52, 55–57, 59–64].

Key differences in risk of bias were seen in domain 2 (risk of bias due to exposure measurement) and domain 5 (risk of bias due to missing data). In domain 2, some studies were ‘high risk’ as they used a narrow or atypical measure of ACEs (*n* = 8) [40, 42, 44, 46, 55, 56, 60, 64]; others were graded as having ‘some concerns’ as they used a broader but still incomplete measure of ACEs (*n* = 8) [43, 45, 48–50, 52, 54, 62]; the remainder were ‘low risk’ as they used an established or comprehensive list of ACE questions [41, 47, 51, 53, 57–59, 61, 63]. In domain 5, some studies were ‘high risk’ as they failed to acknowledge or appropriately address missing data (*n* = 7) [40, 42, 43, 45, 51, 53, 60]; others were graded as having ‘some concerns’ as they had a significant amount of missing data (> 10% for exposure, outcome, or confounders) but mitigated for this with appropriate strategies (*n* = 6) [41, 50, 56, 57, 62, 64]; the remainder were ‘low risk’ as they reported low levels of missing data (*n* = 12) [44, 46–49, 52, 54, 55, 58, 59, 61, 63].

Most studies assessed an exposure that was ‘adverse childhood experiences’ (*n* = 10) [41, 42, 50, 51, 53, 57, 58, 61, 63, 64], ‘childhood maltreatment’ (*n* = 6) [44–46, 48, 49, 59], or ‘childhood adversity’ (*n* = 3) [47, 54, 62]. The other exposures studied were ‘birth phase relative to World War Two’ [40], ‘childhood abuse’ [43], ‘childhood disadvantage’ [56], ‘childhood racial discrimination’ [55], ‘childhood trauma’ [52], and ‘quality of childhood’ (all *n* = 1) [60]. More than half of studies (*n* = 13) did not provide a formal definition of their exposure of choice [42–45, 49, 52–54, 57, 58, 60, 61, 64]. The upper age limit for childhood ranged from < 15 to < 18 years with the most common cut-off being < 18 years (*n* = 9). The median number of ACEs measured in each study was 7 (IQR 4–10). In total, 58 different ACEs were reported; 17 ACEs were reported by at least three studies, whilst 33 ACEs were reported by only one study. The most frequently reported ACEs were physical abuse (*n* = 19) and sexual abuse (*n* = 16) (Table 1). The exposure details for each study can be found in Additional File 1: Table S4.

**Table 1:** Commonly measured adverse childhood experiences

Adverse childhood experience	Number of studies	Studies
Physical abuse	19	Atkinson 2021 [41]; Cromer 2006 [43]; England-Mason 2018 [44]; Godin 2023 [45]; Hanlon 2020 [46]; Hosang 2017 [48]; Hosang 2018 [49]; Lin 2021 [50]; Mendizabal 2022 [51]; Noteboom 2021 [52]; Patterson 2014 [53]; Post 2013 [54]; Sheikh 2018 [56]; Sinnott 2015 [57]; Sosnowski 2022 [58]; Stapp 2020 [59]; Vasquez 2019 [61]; Yang 2021 [62]; Zak-Hunter 2023 [63]
Sexual abuse	16	Atkinson 2021 [41]; Cromer 2006 [43]; England-Mason 2018 [44]; Godin 2023 [45]; Hanlon 2020 [46]; Hosang 2017 [48]; Hosang 2018 [49]; Mendizabal 2022 [51]; Noteboom 2021 [52]; Patterson 2014 [53]; Post 2013 [54]; Sinnott 2015 [57]; Sosnowski 2022 [58]; Stapp 2020 [59]; Vasquez 2019 [61]; Zak-Hunter 2023 [63]
Emotional abuse	12	Atkinson 2021 [41]; Godin 2023 [45]; Hanlon 2020 [46]; Hosang 2017 [48]; Hosang 2018 [49]; Mendizabal 2022 [51]; Noteboom 2021 [52]; Sinnott 2015 [57]; Sosnowski 2022 [58]; Stapp 2020 [59]; Vasquez 2019 [61]; Zak-Hunter 2023 [63]
Emotional neglect	12	Godin 2023 [45]; Hosang 2017 [48]; Hosang 2018 [49]; Lin 2021 [50]; Mendizabal 2022 [51]; Patterson 2014 [53]; Sinnott 2015 [57]; Sosnowski 2022 [58]; Stapp 2020 [59]; Vasquez 2019 [61]; Yang 2021 [62]; Zak-Hunter 2023 [63]
Domestic abuse/violence	11	Atkinson 2021 [41]; England-Mason 2018 [44]; Lin 2021 [50]; Mendizabal 2022 [51]; Patterson 2014 [53]; Sinnott 2015 [57]; Sosnowski 2022 [58]; Vasquez 2019 [61]; Zak-Hunter 2023 [63]; Yang 2021 [62]; Zheng 2022 [64]
Physical neglect	10	Godin 2023 [45]; Hosang 2017 [48]; Hosang 2018 [49]; Mendizabal 2022 [51]; Patterson 2014 [53]; Sinnott 2015 [57]; Sosnowski 2022 [58]; Stapp 2020 [59]; Vasquez 2019 [61]; Zak-Hunter 2023 [63]
Parental divorce/separation	9	Atkinson 2021 [41]; Chandrasekar 2023 [42]; Henchoz 2019 [47]; Lin 2021 [50]; Mendizabal 2022 [51]; Patterson 2014 [53]; Sinnott 2015 [57]; Vasquez 2019 [61]; Zak-Hunter 2023 [63]
Parental mental illness	8	Chandrasekar 2023 [42]; Mendizabal 2022 [51]; Patterson 2014 [53]; Post 2013 [54]; Sinnott 2015 [57]; Sosnowski 2022 [58]; Vasquez 2019 [61]; Yang 2021 [62]
Parental death	7	Atkinson 2021 [41]; Chandrasekar 2023 [42]; Lin 2021 [50]; Sosnowski 2022 [58]; Vasquez 2019 [61]; Yang 2021 [62]; Zheng 2022 [64]
Household member imprisonment	4	Lin 2021 [50]; Patterson 2014 [53]; Sosnowski 2022 [58]; Zak-Hunter 2023 [63]
Parental alcohol/substance misuse	4	Henchoz 2019 [47]; Mendizabal 2022 [51]; Sinnott 2015 [57]; Sosnowski 2022 [58]
Bullying	3	Lin 2021 [50]; Sosnowski 2022 [58]; Zheng 2022 [64]
Household economic environment	3	Henchoz 2019 [47]; Sheikh 2018 [56]; Sosnowski 2022 [58]
Household member mental illness	3	Atkinson 2021 [41]; Lin 2021 [50]; Zak-Hunter 2023 [63]
Parental imprisonment	3	Mendizabal 2022 [51]; Sinnott 2015 [57]; Vasquez 2019 [61]
Parental substance misuse	3	Post 2013 [54]; Vasquez 2019 [61]; Zak-Hunter 2023 [63]
Psychological abuse	3	Noteboom 2021 [52]; Patterson 2014 [53]; Sheikh 2018 [56]

Most commonly measured adverse childhood experiences (ACEs) in the 25 included studies. Only ACEs included in ≥ 3 studies are listed

**Fig. 2:**
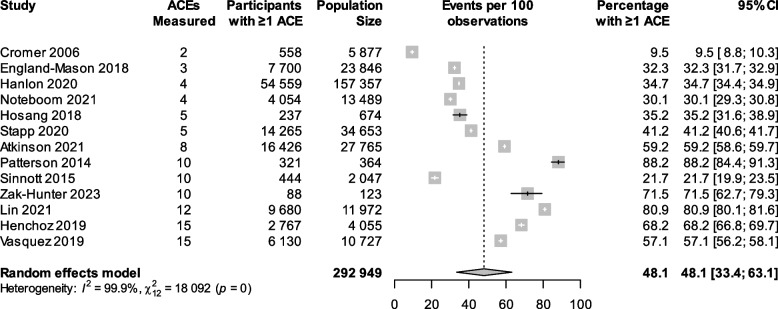
Meta-analysis of prevalence of exposure to ≥ 1 adverse childhood experiences. Meta-analysis of prevalence of exposure to ≥ 1 adverse childhood experience. ACE, adverse childhood experience; CI, confidence interval

Thirteen studies provided sufficient data to allow for a meta-analysis of the prevalence of exposure to ≥ 1 ACE; the pooled prevalence was 48.1% (95% CI 33.4 to 63.1%, *I*^2^ = 99.9%, Cochran *Q* = 18,092, *p* < 0.001) (Fig. 2) [41, 43, 44, 46, 47, 49, 50, 52, 53, 57, 59, 61, 63]. Six studies provided sufficient data to allow for a meta-analysis of the prevalence of exposure to ≥ 4 ACEs; the pooled prevalence was 12.3% (95% CI 3.5 to 35.4%, *I*^2^ = 99.9%, Cochran *Q* = 9071, *p* < 0.001) (Additional File 1: Fig. S1) [46, 50, 51, 53, 59, 63].

Thirteen studies explicitly assessed multimorbidity as an outcome, and all of these defined the threshold for multimorbidity as the presence of two or more LTCs [40–42, 44, 46, 47, 50, 55, 57, 60–62, 64]. The remaining studies assessed comorbidities, morbidity, or disease counts [43, 45, 48, 49, 51–54, 56, 58, 59, 63]. The median number of LTCs measured in each study was 14 (IQR 12–21). In total, 115 different LTCs were reported; 36 LTCs were reported by at least three studies, whilst 63 LTCs were reported by only one study. Two studies did not report the specific LTCs that they measured [51, 53]. The most frequently reported LTCs were hypertension (*n* = 22) and diabetes (*n* = 19) (Table 2). Fourteen studies included at least one mental health LTC. The outcome details for each study can be found in Additional File 1: Table S5.

**Table 2:** Commonly measured long-term conditions

Long-term condition	Number of studies	Studies
Hypertension	22	Arshadipour 2022 [40]; Atkinson 2021 [41]; Chandrasekar 2023 [42]; Cromer 2006 [43]; England-Mason 2018 [44]; Godin 2023 [45]; Hanlon 2020 [46]; Henchoz 2019 [47]; Hosang 2017 [48]; Hosang 2018 [49]; Lin 2021 [50]; Post 2013 [54]; Reyes-Ortiz 2023 [55]; Sheikh 2018 [56]; Sinnott 2015 [57]; Sosnowski 2022 [58]; Stapp 2020 [59]; Tomasdottir 2015 [60]; Vasquez 2019 [61]; Yang 2021 [62]; Zak-Hunter 2023 [63]; Zheng 2022 [64]
Diabetes	21	Arshadipour 2022 [40]; Atkinson 2021 [41]; Chandrasekar 2023 [42]; Cromer 2006 [43]; England-Mason 2018 [44]; Godin 2023 [45]; Hanlon 2020 [46]; Henchoz 2019 [47]; Hosang 2017 [48]; Hosang 2018 [49]; Lin 2021 [50]; Post 2013 [54]; Reyes-Ortiz 2023 [55]; Sheikh 2018 [56]; Sinnott 2015 [57]; Sosnowski 2022 [58]; Tomasdottir 2015 [60]; Vasquez 2019 [61]; Yang 2021 [62]; Zak-Hunter 2023 [63]; Zheng 2022 [64]
Cancer	17	Arshadipour 2022 [40]; Atkinson 2021 [41]; Chandrasekar 2023 [42]; Cromer 2006 [43]; England-Mason 2018 [44]; Godin 2023 [45]; Hanlon 2020 [46]; Henchoz 2019 [47]; Lin 2021 [50]; Post 2013 [54]; Reyes-Ortiz 2023 [55]; Sinnott 2015 [57]; Sosnowski 2022 [58]; Tomasdottir 2015 [60]; Vasquez 2019 [61]; Yang 2021 [62]; Zheng 2022 [64]
Stroke and transient ischaemic attack	17	Arshadipour 2022 [40]; Atkinson 2021 [41]; Chandrasekar 2023 [42]; Cromer 2006 [43]; England-Mason 2018 [44]; Godin 2023 [45]; Hanlon 2020 [46]; Henchoz 2019 [47]; Lin 2021 [50]; Post 2013 [54]; Reyes-Ortiz 2023 [55]; Sheikh 2018 [56]; Sinnott 2015 [57]; Sosnowski 2022 [58]; Vasquez 2019 [61]; Yang 2021 [62]; Zheng 2022 [64]
Asthma	15	Cromer 2006 [43]; England-Mason 2018 [44]; Godin 2023 [45]; Hanlon 2020 [46]; Henchoz 2019 [47]; Hosang 2017 [48]; Hosang 2018 [49]; Lin 2021 [50]; Post 2013 [54]; Sheikh 2018 [56]; Sinnott 2015 [57]; Tomasdottir 2015 [60]; Yang 2021 [62]; Zak-Hunter 2023 [63]; Zheng 2022 [64]
Heart disease	15	Arshadipour 2022 [40]; Atkinson 2021 [41]; Cromer 2006 [43]; England-Mason 2018 [44]; Henchoz 2019 [47]; Hosang 2017 [48]; Hosang 2018 [49]; Lin 2021 [50]; Post 2013 [54]; Sinnott 2015 [57]; Stapp 2020 [59]; Vasquez 2019 [61]; Yang 2021 [62]; Zak-Hunter 2023 [63]; Zheng 2022 [64]
Kidney disease	13	Arshadipour 2022 [40]; Atkinson 2021 [41]; Chandrasekar 2023 [42]; Cromer 2006 [43]; Godin 2023 [45]; Hanlon 2020 [46]; Lin 2021 [50]; Post 2013 [54]; Sheikh 2018 [56]; Sosnowski 2022 [58]; Tomasdottir 2015 [60]; Yang 2021 [62]; Zheng 2022 [64]
Arthritis	12	Cromer 2006 [43]; England-Mason 2018 [44]; Henchoz 2019 [47]; Hosang 2017 [48]; Hosang 2018 [49]; Lin 2021 [50]; Post 2013 [54]; Reyes-Ortiz 2023 [55]; Stapp 2020 [59]; Vasquez 2019 [61]; Yang 2021 [62]; Zheng 2022 [64]
Chronic obstructive pulmonary disease	9	Atkinson 2021 [41]; Cromer 2006 [43]; Hanlon 2020 [46]; Hosang 2017 [48]; Reyes-Ortiz 2023 [55]; Sheikh 2018 [56]; Sinnott 2015 [57]; Tomasdottir 2015 [60]; Vasquez 2019 [61]
Epilepsy and seizures	9	Atkinson 2021 [41]; Chandrasekar 2023 [42]; England-Mason 2018 [44]; Godin 2023 [45]; Hanlon 2020 [46]; Hosang 2017 [48]; Hosang 2018 [49]; Post 2013 [54]; Tomasdottir 2015 [60]
Liver disease	9	Arshadipour 2022 [40]; Cromer 2006 [43]; Hanlon 2020 [46]; Lin 2021 [50]; Post 2013 [54]; Sosnowski 2022 [58]; Stapp 2020 [59]; Yang 2021 [62]; Zheng 2022 [64]
Osteoporosis	9	Atkinson 2021 [41]; Hanlon 2020 [46]; Henchoz 2019 [47]; Hosang 2017 [48]; Hosang 2018 [49]; Reyes-Ortiz 2023 [55]; Sheikh 2018 [56]; Sinnott 2015 [57]; Tomasdottir 2015 [60]
Respiratory disease	8	Arshadipour 2022 [40]; Chandrasekar 2023 [42]; Henchoz 2019 [47]; Lin 2021 [50]; Noteboom 2021 [52]; Sosnowski 2022 [58]; Yang 2021 [62]; Zheng 2022 [64]
Depression	7	Arshadipour 2022 [40]; Chandrasekar 2023 [42]; England-Mason 2018 [44]; Hanlon 2020 [46]; Henchoz 2019 [47]; Sinnott 2015 [57]; Zak-Hunter 2023 [63]
Gastrointestinal disease	7	Arshadipour 2022 [40]; Chandrasekar 2023 [42]; Lin 2021 [50]; Noteboom 2021 [52]; Yang 2021 [62]; Zak-Hunter 2023 [63]; Zheng 2022 [64]
Headache/migraine	7	Atkinson 2021 [41]; England-Mason 2018 [44]; Godin 2023 [45]; Hanlon 2020 [46]; Noteboom 2021 [52]; Post 2013 [54]; Sheikh 2018 [56]
Anxiety	6	Arshadipour 2022 [40]; Atkinson 2021 [41]; England-Mason 2018 [44]; Hanlon 2020 [46]; Sinnott 2015 [57]; Vasquez 2019 [61]
Dyslipidaemia	6	Chandrasekar 2023 [42]; Godin 2023 [45]; Lin 2021 [50]; Tomasdottir 2015 [60]; Yang 2021 [62]; Zheng 2022 [64]
Multiple sclerosis	6	Atkinson 2021 [41]; Godin 2023 [45]; Hanlon 2020 [46]; Hosang 2017 [48]; Hosang 2018 [49]; Post 2013 [54]
Myocardial infarction	6	Atkinson 2021 [41]; Godin 2023 [45]; Sheikh 2018 [56]; Sinnott 2015 [57]; Stapp 2020 [59]; Vasquez 2019 [61]
Thyroid disease	6	Cromer 2006 [43]; Godin 2023 [45]; Hanlon 2020 [46]; Noteboom 2021 [52]; Sinnott 2015 [57]; Tomasdottir 2015 [60]
Angina	5	Atkinson 2021 [41]; Sheikh 2018 [56]; Sinnott 2015 [57]; Stapp 2020 [59]; Vasquez 2019 [61]
Coronary heart disease	5	Chandrasekar 2023 [42]; Godin 2023 [45]; Hanlon 2020 [46]; Henchoz 2019 [47]; Reyes-Ortiz 2023 [55]
Mental health/psychiatric disease	5	Lin 2021 [50]; Reyes-Ortiz 2023 [55]; Tomasdottir 2015 [60]; Yang 2021 [62]; Zheng 2022 [64]
Parkinson's disease	5	Atkinson 2021 [41]; Chandrasekar 2023 [42]; Hanlon 2020 [46]; Henchoz 2019 [47]; Post 2013 [54]
Rheumatoid arthritis	5	Chandrasekar 2023 [42]; Cromer 2006 [43]; Godin 2023 [45]; Sinnott 2015 [57]; Tomasdottir 2015 [60]
Dementia and memory-related disease	4	Hanlon 2020 [46]; Lin 2021 [50]; Yang 2021 [62]; Zheng 2022 [64]
Gastrointestinal ulcer	4	Cromer 2006 [43]; Godin 2023 [45]; Henchoz 2019 [47]; Stapp 2020 [59]
Osteoarthritis	4	Atkinson 2021 [41]; Chandrasekar 2023 [42]; Sinnott 2015 [57]; Tomasdottir 2015 [60]
Allergy	3	Godin 2023 [45]; Post 2013 [54]; Zak-Hunter 2023 [63]
Back pain	3	England-Mason 2018 [44]; Sinnott 2015 [57]; Tomasdottir 2015 [60]
Chronic fatigue syndrome	3	England-Mason 2018 [44]; Hanlon 2020 [46]; Post 2013 [54]
Obesity	3	Chandrasekar 2023 [42]; Sosnowski 2022 [58]; Tomasdottir 2015 [60]
Other long-term condition	3	Cromer 2006 [43]; Post 2013 [54]; Zak-Hunter 2023 [63]
Peripheral vascular disease	3	Atkinson 2021 [41]; Hanlon 2020 [46]; Sinnott 2015 [57]
Substance misuse	3	England-Mason 2018 [44]; Hanlon 2020 [46]; Vasquez 2019 [61]

Most commonly measured long-term conditions in the 25 included studies. Only LTCs included in ≥ 3 studies are listed

**Fig. 3:**
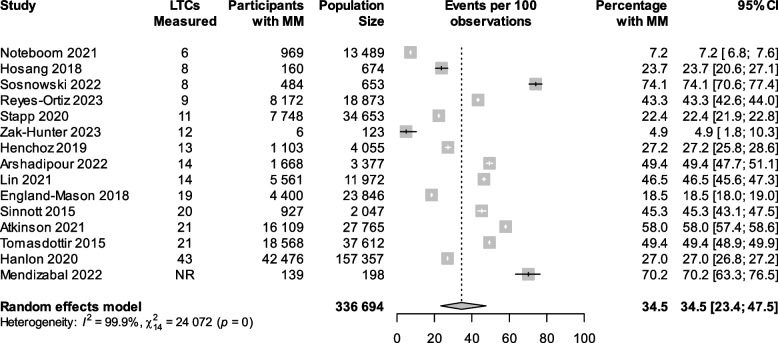
Meta-analysis of prevalence of multimorbidity. Meta-analysis of prevalence of multimorbidity. CI, confidence interval; LTC, long-term condition; MM, multimorbidity

Fifteen studies provided sufficient data to allow for a meta-analysis of the prevalence of multimorbidity; the pooled prevalence was 34.5% (95% CI 23.4 to 47.5%, *I*^2^ = 99.9%, Cochran *Q* = 24,072, *p* < 0.001) (Fig. 3) [40, 41, 44, 46, 47, 49–52, 55, 57–60, 63].

All studies reported significant positive associations between measures of ACE and multimorbidity, though they varied in their means of analysis and reporting of the relationship. Nine studies reported an association between the number of ACEs (variably considered as a continuous or categorical parameter) and multimorbidity [41, 43, 46, 47, 50, 56, 57, 61, 64]. Eight studies reported an association between the number of ACEs and comorbidity counts in specific patient populations [45, 48, 49, 51, 53, 58, 59, 63]. Six studies reported an association between individual ACEs or ACE subgroups and multimorbidity [42–44, 47, 55, 62]. Two studies incorporated a measure of frequency within their ACE measurement tool and reported an association between this ACE score and multimorbidity [52, 54]. Two studies reported an association between proxy measures for ACEs and multimorbidity; one reported ‘birth phase relative to World War Two’, and the other reported a self-report on the overall quality of childhood [40, 60].

**Fig. 4:**
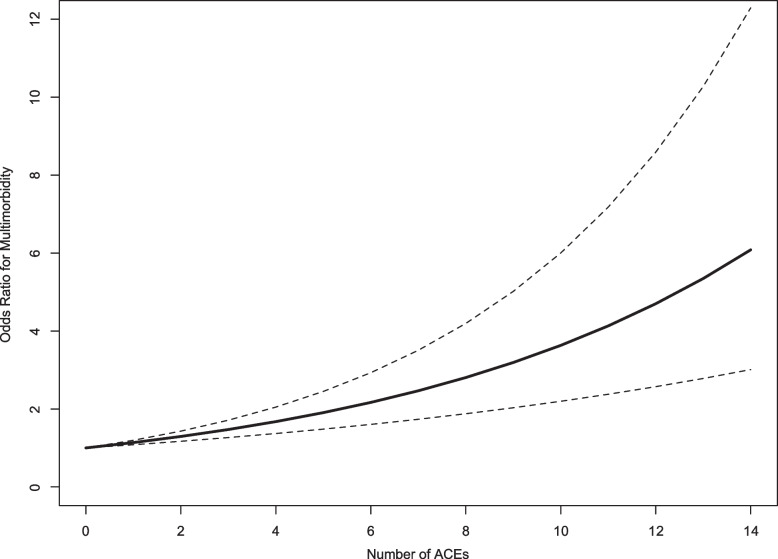
Dose–response meta-analysis of the relationship between adverse childhood experiences and multimorbidity. Dose–response meta-analysis of the relationship between adverse childhood experiences and multimorbidity. Solid black line represents the estimated relationship; dotted black lines represent the 95% confidence intervals for this estimate. ACE, adverse childhood experience

Eight studies, involving a total of 197,981 participants, provided sufficient data (either in the primary text, or following author correspondence) for quantitative synthesis [41, 46, 47, 49–51, 57, 58]. Log-linear (Fig. 4) and non-linear (Additional File 1: Fig. S2) random effects models were compared for goodness of fit: the Wald-type test for linearity was non-significant (*χ*^2^ = 3.7, *p* = 0.16) and the AIC was lower for the linear model (− 7.82 vs 15.86) indicating that the log-linear assumption was valid. There was a significant dose-dependent relationship between ACE exposure and multimorbidity (*p* < 0.001), with every additional ACE exposure contributing to a 12.9% (95% CI 7.9 to 17.9%) increase in the odds for multimorbidity (*I*^2^ = 76.9%, Cochran *Q* = 102, *p* < 0.001).

## Discussion

This systematic review and meta-analysis synthesised the literature on ACEs and multimorbidity and showed a dose-dependent relationship across a large number of participants. Each additional ACE exposure contributed to a 12.9% (95% CI 7.9 to 17.9%) increase in the odds for multimorbidity. This adds to previous meta-analyses that have shown an association between ACEs and individual LTCs, health behaviours, and other health outcomes [1, 28, 31, 65, 66]. However, we also identified substantial inter-study heterogeneity that is likely to have arisen due to variation in the definitions, methodology, and analysis of the included studies, and so our results should be interpreted with these limitations in mind.

Although 25 years have passed since the landmark Adverse Childhood Experiences Study by Felitti et al. [3], there is still no consistent approach to determining what constitutes an ACE. This is reflected in this review, where fewer than half of the 58 different ACEs (*n* = 25, 43.1%) were reported by more than one study and no study reported more than 15 ACEs. Even ACE types that are commonly included are not always assessed in the same way [67], and furthermore, the same question can be interpreted differently in different contexts (e.g. physical punishment for bad behaviour was socially acceptable 50 years ago but is now considered physical abuse in the UK). Although a few validated questionnaires exist, they often focus on a narrow range of ACEs; for example, the childhood trauma questionnaire demonstrates good reliability and validity but focuses on interpersonal ACEs, missing out on household factors (e.g. parental separation), and community factors (e.g. bullying) [68]. Many studies were performed on pre-existing research cohorts or historic healthcare data, where the study authors had limited or no influence on the data collected. As a result, very few individual studies reported on the full breadth of potential ACEs.

ACE research is often based on ACE counts, where the types of ACEs experienced are summed into a single score that is taken as a proxy measure of the burden of childhood stress. The original Adverse Childhood Experiences Study by Felitti et al. took this approach [3], as did 17 of the studies included in this review and our own quantitative synthesis. At the population level, there are benefits to this: ACE counts provide quantifiable and comparable metrics, they are easy to collect and analyse, and in many datasets, they are the only means by which an assessment of childhood stress can be derived. However, there are clear limitations to this method when considering experiences at the individual level, not least the inherent assumptions that different ACEs in the same person are of equal weight or that the same ACE in different people carries the same burden of childhood stress. This limitation was strongly reinforced by our patient and public involvement group (CPAG). Two studies in this review incorporated frequency within their ACE scoring system [52, 54], which adds another dimension to the assessment, but this is insufficient to understand and quantify the ‘impact’ of an ACE within an epidemiological framework.

The definitions of multimorbidity were consistent across the relevant studies but the contributory long-term conditions varied. Fewer than half of the 115 different LTCs (*n* = 52, 45.2%) were reported by more than one study. Part of the challenge is the classification of healthcare conditions. For example, myocardial infarction is commonly caused by coronary heart disease, and both are a form of heart disease. All three were reported as LTCs in the included studies, but which level of pathology should be reported? Mental health LTCs were under-represented within the condition list, with just over half of the included studies assessing at least one (*n* = 14, 56.0%). Given the strong links between ACEs and mental health, and the impact of mental health on quality of life, this is an area for improvement in future research [31, 32]. A recent Delphi consensus study by Ho et al. may help to address these issues: following input from professionals and members of the public they identified 24 LTCs to ‘always include’ and 35 LTCs to ‘usually include’ in multimorbidity research, including nine mental health conditions [9].

As outlined in the introduction, there is a strong evidence base supporting the link between ACEs and long-term health outcomes, including specific LTCs. It is not unreasonable to extrapolate this association to ACEs and multimorbidity, though to our knowledge, the pathophysiological processes that link the two have not been precisely identified. However, similar lines of research are being independently followed in both fields and these areas of overlap may suggest possible mechanisms for a relationship. For example, both ACEs and multimorbidity have been associated with markers of accelerated epigenetic ageing [69, 70], mitochondrial dysfunction [71, 72], and inflammation [22, 73]. More work is required to better understand how these concepts might be linked.

This review used data from a large participant base, with information from 372,162 people contributing to the systematic review and information from 197,981 people contributing to the dose–response meta-analysis. Data from the included studies originated from a range of sources, including healthcare settings and dedicated research cohorts. We believe this is of a sufficient scale and variety to demonstrate the nature and magnitude of the association between ACEs and multimorbidity in these populations.

However, there are some limitations. Firstly, although data came from 11 different countries, only two of those were from outside Europe and North America, and all were from either high- or middle-income countries. Data on ACEs from low-income countries have indicated a higher prevalence of any ACE exposure (consistently > 70%) [74, 75], though how well this predicts health outcomes in these populations is unknown.

Secondly, studies in this review utilised retrospective participant-reported ACE data and so are at risk of recall and reporting bias. Studies utilising prospective assessments are rare and much of the wider ACE literature is open to a similar risk of bias. To date, two studies have compared prospective and retrospective ACE measurements, demonstrating inconsistent results [76, 77]. However, these studies were performed in New Zealand and South Africa, two countries not represented by studies in our review, and had relatively small sample sizes (1037 and 1595 respectively). It is unclear whether these are generalisable to other population groups.

Thirdly, previous research has indicated a close relationship between ACEs and childhood socio-economic status (SES) [78] and between SES and multimorbidity [10, 79]. However, the limitations of the included studies meant we were unable to separate the effect of ACEs from the effect of childhood SES on multimorbidity in this review. Whilst two studies included childhood SES as covariates in their models, others used measures from adulthood (such as adulthood SES, income level, and education level) that are potentially influenced by ACEs and therefore increase the risk of bias due to confounding (Additional File 1: Table S3). Furthermore, as for ACEs and multimorbidity, there is no consistently applied definition of SES and different measures of SES may produce different apparent effects [80]. The complex relationships between ACEs, childhood SES, and multimorbidity remain a challenge for research in this field.

Fourthly, there was a high degree of heterogeneity within included studies, especially relating to the definition and measurement of ACEs and multimorbidity. Whilst this suggests that our results should be interpreted with caution, it is reassuring to see that our meta-analysis of prevalence estimates for exposure to any ACE (48.1%) and multimorbidity (34.5%) are in line with previous estimates in similar populations [2, 11]. Furthermore, we believe that the quantitative synthesis of these relatively heterogenous studies provides important benefit by demonstrating a strong dose–response relationship across a range of contexts.

Our results strengthen the evidence supporting the lasting influence of childhood conditions on adult health and wellbeing. How this understanding is best incorporated into routine practice is still not clear. Currently, the lack of consistency in assessing ACEs limits our ability to understand their impact at both the individual and population level and poses challenges for those looking to incorporate a formalised assessment. Whilst most risk factors for disease (e.g. blood pressure) are usually only relevant within healthcare settings, ACEs are relevant to many other sectors (e.g. social care, education, policing) [81–84], and so consistency of assessment across society is both more important and more challenging to achieve.

Some have suggested that the evidence for the impact of ACEs is strong enough to warrant screening, which would allow early identification of potential harms to children and interventions to prevent them. This approach has been implemented in California, USA [85–87]. However, this is controversial, and others argue that screening is premature with the current evidence base [88–90]. Firstly, not everyone who is exposed to ACEs develops poor health outcomes, and it is not clear how to identify those who are at highest risk. Many people appear to be vulnerable, with more adverse health outcomes following ACE exposure than those who are not exposed, whilst others appear to be more resilient, with good health in later life despite multiple ACE exposures [91] It may be that supportive environments can mitigate the long-term effects of ACE exposure and promote resilience [92, 93]. Secondly, there are no accepted interventions for managing the impact of an identified ACE. As identified above, different ACEs may require input from different sectors (e.g. healthcare, social care, education, police), and so collating this evidence may be challenging. At present, ACEs screening does not meet the Wilson-Jungner criteria for a screening programme [94].

Existing healthcare systems are poorly designed to deal with the complexities of addressing ACEs and multimorbidity. Possibly, ways to improve this might be allocating more time per patient, prioritising continuity of care to foster long-term relationships, and greater integration between different healthcare providers (most notably primary vs secondary care teams, or physical vs mental health teams). However, such changes often demand additional resources (e.g. staff, infrastructure, processes), which are challenging to source when existing healthcare systems are already stretched [95, 96]. Nevertheless, increasing the spotlight on ACEs and multimorbidity may help to focus attention and ultimately bring improvements to patient care and experience.

## Conclusions

ACEs are associated with a range of poor long-term health outcomes, including harmful health behaviours and individual long-term conditions. Multimorbidity is becoming more common as global populations age, and it increases the complexity and cost of healthcare provision. This is the first systematic review and meta-analysis to synthesise the literature on ACEs and multimorbidity, showing a statistically significant dose-dependent relationship across a large number of participants, albeit with a high degree of inter-study heterogeneity. This consolidates and enhances an increasing body of data supporting the role of ACEs in determining long-term health outcomes. Whilst these observational studies do not confirm causality, the weight and consistency of evidence is such that we can be confident in the link. The challenge for healthcare practitioners, managers, policymakers, and governments is incorporating this body of evidence into routine practice to improve the health and wellbeing of our societies.

### Supplementary Information

Additional File 1: Tables S1-S5 and Figures S1-S2. Table S1: Search strategy, Table S2: Characteristics of studies included in the systematic review, Table S3: Risk of bias assessment (ROBINS-E), Table S4: Exposure details (adverse childhood experiences), Table S5: Outcome details (multimorbidity), Figure S1: Meta-analysis of prevalence of exposure to ≥4 adverse childhood experiences, Figure S2: Dose-response meta-analysis of the relationship between adverse childhood experiences and multimorbidity (using a non-linear/restricted cubic spline model).

## Data Availability

No additional data was generated for this review. The data used were found in the referenced papers or provided through correspondence with the study authors.

## Abbreviations

ACE: Adverse childhood experience
AIC: Akaike information criterion
CAPE: CONSORTIUM Against pain inequality
CI: Confidence interval
CPAG: Chronic pain advisory group
IQR: Interquartile range
LTC: Long-term condition
PROSPERO: International prospective register of systematic reviews
PRISMA: Preferred reporting items for systematic reviews and meta-analyses
ROBINS-E: Risk of bias in non-randomised studies of exposures
SES: Socio-economic status
