# Supplementary material for: The impact of adverse childhood experiences on multimorbidity: a systematic review and meta-analysis
Source: BMC Med. 2024 Aug 15;22:315. doi: 10.1186/s12916-024-03505-w (PMC11325707; doi:10.1186/s12916-024-03505-w)
Supplement: Supplementary file 1 — Additional File 1: Tables S1-S5 and Figures S1-S2. Table S1: Search strategy, Table S2: Characteristics of studies included in the systematic review, Table S3: Risk of bias assessment (ROBINS-E), Table S4: Exposure details (adverse childhood experiences), Table S5: Outcome details (multimorbidity), Figure S1: Meta-analysis of prevalence of exposure to ≥4 adverse childhood experiences, Figure S2: Dose-response meta-analysis of the relationship between adverse childhood experiences and multimorbidity (using a non-linear/restricted cubic spline model). [file 12916_2024_3505_MOESM1_ESM.docx]

# The impact of adverse childhood experiences on multimorbidity: a systematic review and meta-analysis

Dhaneesha N.S. Senaratne, Bhushan Thakkar, Blair H. Smith, Tim G. Hales, Louise Marryat, Lesley A. Colvin

# Additional File 1: Supplementary Materials: Table of Contents

[Table S1: Search strategy 2](#_Toc152330648)

[Table S2: Characteristics of studies included in the systematic review 3](#_Toc152330649)

[Table S3: Risk of bias assessment (ROBINS-E) 5](#_Toc152330650)

[Table S4: Exposure details (adverse childhood experiences) 6](#_Toc152330651)

[Table S5: Outcome details (multimorbidity) 11](#_Toc152330652)

[Figure S1: Meta-analysis of prevalence of exposure to ≥4 adverse childhood experiences 18](#_Toc152330653)

[Figure S2: Dose-response meta-analysis of the relationship between adverse childhood experiences and multimorbidity (using a non-linear/restricted cubic spline model) 19](#_Toc152330654)

Table S1: Search strategy

| # | APA PsycNET | CINAHL Plus via EBSCO | Cochrane CENTRAL | Embase via Ovid | MEDLINE via EBSCO | Scopus | Web of Science |
| --- | --- | --- | --- | --- | --- | --- | --- |
| 1 | (adverse childhood experiences) OR (advers* NEAR/2 child* NEAR/2 experienc*) | (adverse childhood experiences) OR (advers* N2 child* N2 experienc*) | (adverse childhood experiences) OR (advers* NEAR/2 child* NEAR/2 experienc*) | (adverse childhood experiences) OR (advers* adj2 child* adj2 experienc*) | (adverse childhood experiences) OR (advers* N2 child* N2 experienc*) | (adverse childhood experiences) OR (advers* w/1 child* w/1 experienc*) | (adverse childhood experiences) OR (advers* NEAR/2 child* NEAR/2 experienc*) |
| 2 | child* NEAR/2 (abus* OR advers* OR maltreat* OR neglect* OR trauma*) | child* N2 (abus* OR advers* OR maltreat* OR neglect* OR trauma*) | child* NEAR/2 (abus* OR advers* OR maltreat* OR neglect* OR trauma*) | child* adj2 (abus* OR advers* OR maltreat* OR neglect* OR trauma*) | child* N2 (abus* OR advers* OR maltreat* OR neglect* OR trauma*) | child* w/1 (abus* OR advers* OR maltreat* OR neglect* OR trauma*) | child* NEAR/2 (abus* OR advers* OR maltreat* OR neglect* OR trauma*) |
| 3 | #1 OR #2 | #1 OR #2 | #1 OR #2 | #1 OR #2 | #1 OR #2 | #1 OR #2 | #1 OR #2 |
| 4 | multimorbidity OR multimorbid* OR multi-morbid* | multimorbidity OR multimorbid* OR multi-morbid* | multimorbidity OR multimorbid* OR multi-morbid* | multimorbidity OR multimorbid* OR multi-morbid* | multimorbidity OR multimorbid* OR multi-morbid* | multimorbidity OR multimorbid* OR multi-morbid* | multimorbidity OR multimorbid* OR multi-morbid* |
| 5 | comorbidity OR comorbid* OR co-morbid* | comorbidity OR comorbid* OR co-morbid* | comorbidity OR comorbid* OR co-morbid* | comorbidity OR comorbid* OR co-morbid* | comorbidity OR comorbid* OR co-morbid* | comorbidity OR comorbid* OR co-morbid* | comorbidity OR comorbid* OR co-morbid* |
| 6 | smultipl* NEAR/2 (long-term OR long term) NEAR/2 (condition* OR disease* OR disorder* OR illness*) | multipl* N2 (long-term OR long term) N2 (condition* OR disease* OR disorder* OR illness*) | multipl* NEAR/2 (long-term OR long term) NEAR/2 (condition* OR disease* OR disorder* OR illness*) | multipl* adj2 (long-term OR long term) adj2 (condition* OR disease* OR disorder* OR illness*) | multipl* N2 (long-term OR long term) N2 (condition* OR disease* OR disorder* OR illness*) | multipl* w/1 (long-term OR "long term") w/1 (condition* OR disease* OR disorder* OR illness*) | multipl* NEAR/2 (long-term OR "long term") NEAR/2 (condition* OR disease* OR disorder* OR illness*) |
| 7 | #4 OR #5 OR #6 | #4 OR #5 OR #6 | #4 OR #5 OR #6 | #4 OR #5 OR #6 | #4 OR #5 OR #6 | #4 OR #5 OR #6 | #4 OR #5 OR #6 |
| 8 | #3 AND #7 | #3 AND #7 | #3 AND #7 | #3 AND #7 | #3 AND #7 | #3 AND #7 | #3 AND #7 |

Table S2: Characteristics of studies included in the systematic review

| First Author & Year | Participant Cohort | Cohort Country | Years of Recruitment | Sample Size | Female Gender (%) | Age |
| --- | --- | --- | --- | --- | --- | --- |
| Arshadipour 2022 | Cooperative Health Research in the Region of Augsburg, waves 1 & 3 (KORA-Age 1, KORA-Age 3): participants aged ≥65 in Augsburg and two adjacent regions. | Germany | 2008-2009 and 2016 | 3377 | 52.2% | Range 65-71; mean 67.9*; SD 1.9* |
| Atkinson 2021 | Canadian Longitudinal Study of Aging (CLSA): a national longitudinal study involving a stratified random sample of community-dwelling participants aged 45-85. | Canada | 2011-2015 | 27765 | 50.9% | Range 45-85 |
| Chandrasekar 2023 | 1946 MRC National Survey of Health and Development: a sample derived from a maternity survey of births during March 1946. | UK | 1982 | 3264 | 50.3% | Not applicable - longitudinal analysis of a birth cohort |
| Cromer 2006 | National Comorbidity Survey: a nationwide epidemiological study. | USA | 1990-1992 | 5877 | NR | Range 15-54; mean 33.2 |
| England-Mason 2018 | Canadian Community Health Survey - Mental Health 2012 (CCHS-MH 2012): a representative sample of respondents aged ≥15 from 10 Canadian provinces. | Canada | 2012 | 23846 | 50.9% | Not reported for complete sample |
| Godin 2023 | Outpatient clinics within the FondaMental Advanced Centers of Expertise in Bipolar Disorders (FACE-BD) network: all patients ≥16 years attending one of 12 centres with a diagnosis of bipolar disorder. | France | 2009-2020 | 2891 | 62.2% | Mean 40.5; SD 12.9 |
| Hanlon 2020 | UK Biobank: adults aged 40-69 recruited from General Practice databases. | UK | 2006-2010 | 157357 | NR | Not reported for complete sample |
| Henchoz 2019 | Lausanne Cohort 65+ Study (Lc65+): randomly selected representative samples of adults aged 65-70 in Lausanne. | Switzerland | 2004, 2009, and 2014 | 4731 | 58.0% | Range 65-70; median 67.8; IQR 2.5; mean 67.9; SD 1.5 |
| Hosang 2017 | Cases recontacted from a previous case control study, control data used from a previous study. | UK | Not reported | 426 | 61.3% | Range 24-72; mean 47.8*; SD 9.2* |
| Hosang 2018 | Bipolar cases recontacted from a previous case control study, unipolar depression data used from a previous study, control data used from a previous study. | UK | Not reported | 674 | 65.9% | Mean 46.9*; SD 10.7* |
| Lin 2021 | China Health and Retirement Longitudinal Study (CHARLS): randomly selected adults from 28 provinces across China. | China | 2011 | 11972 | 51.6% | Mean 59.9; SD 9.6 |
| Mendizabal 2022 | Adults attending an outpatient neurology clinic at the University of Pennsylvania. | USA | 2019-2020 | 198 | 55.6% | Not reported for complete sample |
| Noteboom 2021 | Netherlands Mental Health Survey and Incidence Study 1 and 2 (NEMESIS-1, NEMESIS-2): a nationally representative sample. | The Netherlands | 1996 and 2007-2009 | 13489 | 54.2% | Range 18-64; mean 42.6; SD 12.5 |
| Patterson 2014 | Vancouver At Home Study: adults ≥19 with no permanent housing. | Canada | 2009-2011 | 364 | 29.9% | Mean 41.1; SD 10.6 |
| Post 2013 | Bipolar Collaborative Network: adults with bipolar disorder recruited from 7 sites. | USA, The Netherlands & Germany | 1995-2002 | 904 | NR | Mean 41 |
| Reyes-Ortiz 2023 | Salud, Bienestar y Envejecimiento (SABE) Colombia Study: a cross-sectional study of adults aged ≥60 in urban and rural Colombia. | Colombia | 2015 | 18873 | 53.6% | Mean 68.4; SD 13.7* |
| Sheikh 2018 | Tromso VI Study: a longitudinal prospective study | Norway | 2007-2008 | 10765 | 51.1% | Range 30-87; mean 57.5; SD 12.6 |
| Sinnott 2015 | The Mitchelstown Cohort | Republic of Ireland | 2010-2011 | 2047 | 50.8% | Range 50-69; mean 55.8*; SD 15.8* |
| Sosnowski 2022 | AIDS Linked to the Intravenous Experience (ALIVE) Study | USA | 1988, 1994-1995, 2005-2008, and 2015-2018 | 653 | 32.6% | Median 48; IQR 42-52 |
| Stapp 2020 | National Epidemiologic Survey on Alcohol and Related Conditions (NESARC), waves 1 & 2: a nationwide nationally representative household survey of adults. | USA | 2001-2002 and 2004-2005 | 34653 | 58.0% | Not reported for complete sample |
| Tomasdottir 2015 | Nord-Trøndelag Health Study (HUNT3) | Norway | 2006-2008 | 37612 | 54.1% | Range 30-69; mean 51.3*; SD 10.6* |
| Vasquez 2019 | National Epidemiological Survey on Alcohol and Related Conditions III (NESARC-III) | USA | 2012-2013 | 10727 | 54.0% | Mean 66.8; SD 11.4* |
| Yang 2021 | China Health and Retirement Longitudinal Study (CHARLS): randomly selected adults from 28 provinces across China. | China | 2011 | 14093 | 52.1% | Range 45-101; mean 59.5 |
| Zak-Hunter 2023 | Family Matters | USA | 2014-2016 | 123 | 100.0% | Mean 31.1*; SD 5.0* |
| Zheng 2022 | China Health and Retirement Longitudinal Study (CHARLS): randomly selected adults from 28 provinces across China. | China | 2011-2012 | 4440 | 55.2% | Mean 58.6; SD 8.8 |

SD = standard deviation. *Value calculated from data available in paper.

Table S3: Risk of bias assessment (ROBINS-E)

| First Author & Year | Domain 1: Confounding | Domain 2: Exposure Measurement | Domain 3: Participant Selection | Domain 4: Post-Exposure Interventions | Domain 5: Missing Data | Domain 6: Outcome Measurement | Domain 7: Selection of Reported Result | Overall |
| --- | --- | --- | --- | --- | --- | --- | --- | --- |
| Arshadipour 2022 | High | High | Some Concerns | Low | High | Low | Low | Very High |
| Atkinson 2021 | High | Low | Some Concerns | Low | Some Concerns | Low | Low | High |
| Chandrasekar 2023 | Low | High | Some Concerns | Low | High | Low | Low | High |
| Cromer 2006 | High | Some Concerns | Some Concerns | Low | High | Low | Low | High |
| England-Mason 2018 | High | High | Some Concerns | Low | Low | Low | Low | High |
| Godin 2023 | High | Some Concerns | High | Low | High | Low | Low | Very High |
| Hanlon 2020 | High | High | Some Concerns | Low | Low | Low | Low | High |
| Henchoz 2019 | High | Low | Some Concerns | Low | Low | Low | Low | High |
| Hosang 2017 | High | Some Concerns | High | Low | Low | Low | Low | High |
| Hosang 2018 | High | Some Concerns | High | Low | Low | Low | Low | High |
| Lin 2021 | High | Some Concerns | Some Concerns | Low | Some Concerns | Low | Low | High |
| Mendizabal 2022 | High | Low | High | Low | High | Low | Low | Very High |
| Noteboom 2021 | High | Some Concerns | Some Concerns | Low | Low | Low | Low | High |
| Patterson 2014 | High | Low | High | Low | High | Low | High | Very High |
| Post 2013 | High | Some Concerns | High | Low | Low | Low | High | Very High |
| Reyes-Ortiz 2023 | High | High | Some Concerns | Low | Low | Low | Low | High |
| Sheikh 2018 | High | High | Some Concerns | Low | Some Concerns | Low | Low | High |
| Sinnott 2015 | High | Low | Some Concerns | Low | Some Concerns | Low | Low | High |
| Sosnowski 2022 | High | Low | High | Low | Low | Low | Low | High |
| Stapp 2020 | High | Low | Some Concerns | Low | Low | Low | Low | High |
| Tomasdottir 2015 | High | High | Some Concerns | Low | High | Low | Low | Very High |
| Vasquez 2019 | High | Low | Some Concerns | Low | Low | Low | High | High |
| Yang 2021 | High | Some Concerns | Some Concerns | Low | Some Concerns | Low | Low | High |
| Zak-Hunter 2023 | High | Low | Some Concerns | Low | Low | Low | Low | High |
| Zheng 2022 | High | High | Some Concerns | Low | Some Concerns | Low | Low | High |

Overall risk of bias rating was determined by the ROBINS-E algorithm. This was usually the highest rating across all domains, however if ≥3 domains shared the highest rating then the overall assessment was upgraded to a higher risk level (e.g. ≥3 “High” ratings in individual domains led to a “Very High” overall rating).

Table S4: Exposure details (adverse childhood experiences)

| First Author & Year | Exposure | Exposure Definition | Exposure Tool | Childhood Upper Age Limit | Number of Adversities | List of Adversities | Adversity Prevalence |
| --- | --- | --- | --- | --- | --- | --- | --- |
| Arshadipour 2022 | Birth phase relative to WW2 | Date of birth relative to WW2. | NA | NA | 1 | date of birth relative to WW2 | NA |
| Atkinson 2021 | Adverse childhood experiences | "potentially traumatic childhood events or environmental aspects that undermine the child’s sense of safety" | 14 questions adapted from the Childhood Experiences of Violence Questionnaire (CEVQ) and the National Longitudinal Study of Adolescent to Adult Health Wave II questionnaire | <16 | 8 | physical abuse; emotional abuse; sexual abuse; neglect; intimate partner violence; parental divorce/separation; parental death; living with a family member with mental health problems | 0: 35.9% 1: 26.9% 2: 15.5% ≥3: 21.8% |
| Chandrasekar 2023 | Adverse childhood experiences | NR | Questions from NSHD database | <15 | 9 | parental death; parental divorce/separation; regular/permanent separation from mother; residential instability; parental physical illness; parental psychiatric illness; severe/chronic parental illness; childhood physical illness; childhood psychiatric illness | NR |
| Cromer 2006 | Childhood abuse | NR | WHO Composite International Diagnostic Interview | <15 | 2 | sexual abuse; physical abuse | 0: 90.5% ≥1: 9.5% |
| England-Mason 2018 | Childhood maltreatment | NR | Questions from the Childhood Experiences of Violence Questionnaire (CEVQ) and from previous surveys administered by Statistics Canada | <16 | 3 | sexual abuse; physical abuse; exposure to intimate partner violence | 0: 66.8% ≥1: 32.3% |
| Godin 2023 | Childhood maltreatment | NR | Childhood Trauma Questionnaire (CTQ) | <18 | 5 | physical abuse; sexual abuse; emotional abuse; physical neglect; emotional neglect | NR |
| Hanlon 2020 | Childhood maltreatment | "all types of physical and/or emotional ill-treatment, sexual abuse, neglect, negligence and commercial or other exploitation resulting in actual or potential harm" | 4/28 questions from Childhood Trauma Questionnaire | NR | 4 | physical abuse; emotional abuse; sexual abuse; neglect | 0: 66.5% 1: 21.9% 2: 8.4% 3: 2.6% 4: 0.6% |
| Henchoz 2019 | Childhood adversity | "a range of potentially difficult or unpleasant situations or experiences, usually before the age of sixteen" | Author derived questions | <16 | 15 | premature birth; food restrictions; child labour; family economic environment; serious illness or accident; death of household member; parental separation; remoteness of parent for >6 months; residing in a boarding house; serious illness or accident of family member; parental alcoholism or drug abuse; parental unemployment or business failure; physical or emotional aggression (including abuse and neglect); change of country or linguistic region; other events | NR |
| Hosang 2017 | Childhood maltreatment | "both abuse (e.g. sexual, emotional and physical abuse) and neglect (lack of provision for the individual’s needs by their caregiver, including food, shelter and support)" | Childhood Trauma Questionnaire (CTQ) | <18 | 5 | physical abuse; sexual abuse; emotional abuse; physical neglect; emotional neglect | 0: 76.3% ≥1: 23.7% |
| Hosang 2018 | Childhood maltreatment | NR | Childhood Trauma Questionnaire (CTQ) | <18 | 5 | sexual abuse; emotional abuse; physical abuse; emotional neglect; physical neglect | NR |
| Lin 2021 | Adverse childhood experiences | "potentially stressful experiences that occur in childhood" | Questions from CHARLS database | <16 | 12 | physical abuse; emotional neglect; household substance abuse; household mental illness; domestic violence; incarcerated household member; parental separation or divorce; unsafe neighborhood; bullying; parental death; sibling death; parental disability | 0: 19.1% 1: 26.9% 2: 21.8% 3: 14.2% ≥4: 18.0% |
| Mendizabal 2022 | Adverse childhood experiences | "traumatic events experienced before age 18 years" | Adverse Childhood Experiences Questionnaire (Felitti 1998) | <18 | 10 | emotional abuse; physical abuse; sexual abuse; emotional neglect; physical neglect; domestic abuse; parents divorced; parents in prison; parental addiction; parental mental illness | ≥4: 23.7% |
| Noteboom 2021 | Childhood trauma | NR | Childhood Trauma NEMESIS Questionnaire | <16 | 4 | emotional abuse; psychological abuse; physical abuse; sexual abuse | 0: 71.1% ≥1: 28.9% |
| Patterson 2014 | Adverse childhood experiences | NR | Adverse Childhood Experiences Questionnaire (Felitti 1998) | <18 | 10 | psychological abuse; physical abuse; sexual abuse; emotional neglect; physical neglect; parental separation or divorce; exposure to substance abuse; parental mental illness; violent treatment of mother or stepmother; incarceration in the household | 0: 11.8% 1: 12.4% 2: 13.7% 3: 11.3% 4: 9.1% ≥5: 41.8% |
| Post 2013 | Childhood adversity | NR | Author derived questions creating childhood adversity score, that incoporates adversity count and frequency | NR | 7 | physical abuse; sexual abuse; verbal abuse; parental affective disorder; parental drug abuse; parental alcohol abuse; parental suicide or suicide attempt | NR |
| Reyes-Ortiz 2023 | Childhood racial discrimination | "discrimination based on skin color" | Question modified from discrimination scales | NR | 1 | childhood racial discrimination | Any: 4.6% |
| Sheikh 2018 | Childhood disadvantage | "a conglomerate of factors that have been used in a similar manner in previous studies" | Author derived questions | NR | 6 | low mother’s education; low father’s education; difficult subjective childhood financial conditions; psychological abuse; physical abuse; substance abuse distress | Mean 1.92; SD 1.22 |
| Sinnott 2015 | Adverse childhood experiences | NR | Adverse Childhood Experiences Questionnaire (Felitti 1998) | ≤18 | 10 | emotional abuse; physical abuse; sexual abuse; emotional neglect; physical neglect; domestic abuse; parents divorced; parents in prison; parental addiction; parental mental illness | 0: 78.3% ≥1: 21.7% |
| Sosnowski 2022 | Adverse childhood experiences | NR | Modified Adverse Childhood Experiences Questionnaire (Finkelhor 2015) | <18 | 14 | physical neglect; emotional neglect; physical abuse; emotional abuse; sexual abuse; loss of a parent; domestic violence; parent substance abuse; parent mental illness; incarceration of a household member; bullying; social ostracization; neighborhood violence; poverty | 0-1: 39.2% 2-4: 33.3% 5-9: 22.5% ≥10: 11.1% |
| Stapp 2020 | Childhood maltreatment | "physical, sexual, or emotional abuse, as well as physical or emotional neglect" | Questions derived from Conflicts Tactics Scale and Childhood Trauma Questionnaire | <18 | 5 | physical neglect; physical abuse; emotional neglect; emotional abuse; sexual abuse | 0: 58.3% 1: 23.7% 2: 8.9% 3: 4.5% 4: 2.7% 5: 1.2% |
| Tomasdottir 2015 | Quality of childhood | NR | Author derived questions | NR | 1 | overall childhood quality | NR |
| Vasquez 2019 | Adverse childhood experiences | NR | Questions derived from Conflicts Tactics Scale and Childhood Trauma Questionnaire, used in previous NESARC waves | <18 | 15 | physical neglect; emotional neglect; physical abuse; emotional abuse; sexual abuse; witnessed domestic violence; parental alcohol misuse; parental drug misuse; parent sent to prison; parent treated/hospitalized for mental illness; parent attempt suicide; parent commit suicide; adoption/foster care/orphanage; parental separation; parental death | 0: 42.9% ≥1: 57.1% |
| Yang 2021 | Childhood adversity | "an exposure to various types of traumatic events or situations during childhood, including sudden distressing events (ie, parental death or divorce), maltreatment (eg, physical, emotional or sexual abuse) and negative living environments (eg, caregiver psychopathology)" | Questions from CHARLS database | <17 | 12 | parental physical abuse; parental emotional neglect; parental mental status; quality of parental relationship; parental death; adequacy of food; father's education; father's occupation | NR |
| Zak-Hunter 2023 | Adverse childhood experiences | "stressful and potentially traumatizing events that occur during childhood" | Adverse Childhood Experiences Questionnaire (Felitti 1998) | <18 | 10 | parental separation; household member mental illness; household member imprisonment; physical neglect; emotional neglect; parental substance misuse; domestic abuse; emotional abuse; physical abuse; sexual abuse | 0: 28% ≥1: 72% |
| Zheng 2022 | Adverse childhood experiences | NR | Questions from CHARLS database | <17 | 12 | had friends; bullied by buddy; relationship with female guardian; hutted by female guardian; relationship with male guardian; hutted by male guardian; parental relationship; parental quarrel; father beat up mother; mother beat up father; mother death; father death | Mean 2.6; SD 1.9 |

NA = not applicable; NR = not reported; SD = standard deviation.

Table S5: Outcome details (multimorbidity)

| First Author & Year | Outcome | Outcome Definition | Outcome Tool | Number of Long-term Conditions | List of Long-term Conditions | Multimorbidity Prevalence |
| --- | --- | --- | --- | --- | --- | --- |
| Arshadipour 2022 | Multimorbidity | "the presence of two or more concomitant chronic diseases in individuals" | Author determined list of "major chronic diseases" based on KORA-Age questionnaire and Charlson Comorbidity Index | 14 | hypertension; eye disease (cataract, retinitis pigmentosa, glaucoma, macular degeneration, diabetic retinopathy); heart disease (myocardial infarction, coronary artery disease, congestive heart failure, coronary heart failure, angina); diabetes; joint disease (arthritis, rheumatism); lung disease (asthma, chronic bronchitis, emphysema); gastrointestinal disease (colitis, cholecystic, gastric, ulcer); stroke; cancer; kidney diseases; liver diseases; neurological diseases (epilepsy, Parkinson's disease, sclerosis); depression; anxiety | 49.4% |
| Atkinson 2021 | Multimorbidity | "co-occurrence of at least two chronic health conditions, with comorbidities unrelated to an index diagnosis" | Author determined list of 21 conditions with "a high prevalence and impact or burden in the population… in North America" | 21 | heart disease; myocardial infarction; angina; stroke; transient ischaemic attack; peripheral vascular disease; hypertension; diabetes; chronic obstructive pulmonary disease; Parkinson's disease; epilepsy; multiple sclerosis; migraines; osteoarthritis; osteoporosis; kidney disease; cataracts; glaucoma; cancer; mood disorders; anxiety disorders | 57.1% |
| Chandrasekar 2023 | Multimorbidity | "≥ 2 health disorders in an individual" | Questions from NSHD database | 18 | obesity; hypertension; dyslipidaemia; diabetes; coronary heart disease; stroke; osteoarthritis; rheumatoid arthritis; anaemia; skin disorders; respiratory disorders; gastrointestinal disorders; kidney disorders; Parkinson's disease; cancer; epilepsy; depression; psychotic disorders | At age 36: 16% At age 69: 80% |
| Cromer 2006 | Health problems | NR | Author determined list of "serious health problems experienced during the last year" | 21 | arthritis; rheumatism; asthma; blindness or deafness; bronchitis; tuberculosis; diabetes; hypertension; heart problems; hernia; kidney disease; liver disease; lupus; thyroid disease; auto-immune disorders; neurological problems; stroke; stomach or gallbladder disease; ulcers; cancer; any other serious health problem | NR |
| England-Mason 2018 | Multimorbidity | "the co-occurrence of two or more chronic health conditions within the same patient" | Author determined list of "questions pertaining to long-term health" | 19 | Physical conditions: asthma; arthritis; high blood pressure; diabetes; epilepsy; heart disease; cancer; stroke; bowel disease; chronic fatigue syndrome  Pain conditions: back pain; migraine; headaches  Mental disorders: major depressive episode; bipolar disorder; generalized anxiety disorder; alcohol abuse/dependence; cannabis abuse/dependence; other drug abuse/dependence | 18.5% |
| Godin 2023 | Medical morbidity | NR | Author determined list of conditions | 29 | headache/migraine; multiple sclerosis; epilepsy; meningitis; stroke; hypertension; coronary disease; myocardial infarction; cardiac dysrhythmia; diabetes; thyroid disorders; dislipidemia; nephropathy; acute retention of urine; psoriasis; eczema; drug-induced toxidermia; acne; cirrhosis; drug-induced hepatitis; peptic ulcer; bowel disease; asthma; allergy; systemic lupus erythematosus; rheumatoid arthritis; cancer; HIV; chronic viral hepatitis | NR |
| Hanlon 2020 | Multimorbidity | "the presence of two or more long-term conditions" | "list of 43 LTCs originally established for a large epidemiological study in Scotland, through systematic review, the Quality and Outcomes Framework, NHS Scotland and an expert panel12 and subsequently amended for UK Biobank" | 43 | painful conditions; hypertension; depression; asthma; coronary heart disease; treated dyspepsia; diabetes; thyroid disorders; rheumatoid arthritis, other inflammatory polyarthropathies, systemic connective tissue disorders and systemic autoimmue disorders; chronic obstructive pulmonary disease; anxiety, other neurotic, stress-related and somatoform disorders; irritable bowel syndrome; alcohol problems; other psychoactive substance abuse; treated constipation; stroke and transient ischaemic attack; chronic kidney disease; diverticular disease of intestine; atrial fibrillation; peripheral vascular disease; heart failure; prostate disorders; glaucoma; epilepsy; dementia; schizophrenia and bipolar disorder; psoriasis or eczema; inflammatory bowel disease; migraine; chronic sinusitus; anorexia or bulimia; bronchiectasis; Parkinson's disease; multiple sclerosis; viral hepatitis; chronic liver disease; osteoporosis; chronic fatigue syndrome; endometriosis; Meniere's disease; pernicious anaemia; polycystic ovary disease; cancer | NR |
| Henchoz 2019 | Multimorbidity | "co-occurrence of two or more chronic diseases or medical conditions" | Author determined list of "13 common health conditions diagnosed by a physician" | 13 | painful conditions; hypertension; depression; asthma; coronary heart disease; treated dyspepsia; diabetes; thyroid disorders; rheumatoid arthritis, other inflammatory polyarthropathies, systemic connective tissue disorders and systemic autoimmue disorders; chronic obstructive pulmonary disease; anxiety, other neurotic, stress-related and somatoform disorders; irritable bowel syndrome; alcohol problems; other psychoactive substance abuse; treated constipation; stroke and transient ischaemic attack; chronic kidney disease; diverticular disease of intestine; atrial fibrillation; peripheral vascular disease; heart failure; prostate disorders; glaucoma; epilepsy; dementia; schizophrenia and bipolar disorder; psoriasis or eczema; inflammatory bowel disease; migraine; chronic sinusitus; anorexia or bulimia; bronchiectasis; Parkinson's disease; multiple sclerosis; viral hepatitis; chronic liver disease; osteoporosis; chronic fatigue syndrome; endometriosis; Meniere's disease; pernicious anaemia; polycystic ovary disease; cancer | 27.4% |
| Hosang 2017 | Medical illnesses | NR | Author determined list of conditions | 10 | heart problems (i.e. stroke, angina and heart attack); asthma; diabetes (I and II); arthritis (i.e. osteoarthritis, rheumatoid arthritis and other types of arthritis); hypertension; epilepsy or convulsions; osteoporosis; multiple sclerosis; emphysema or chronic bronchitis; post herpetic neuralgia | 32.2% |
| Hosang 2018 | Medical illnesses | NR | Author determined list of conditions | 8 | heart problems (stroke, angina and heart attack); asthma; diabetes (type 1 and type 2); arthritis (osteoarthritis and rheumatoid arthritis); hypertension; epilepsy or convulsions; osteoporosis; multiple sclerosis | 34.9% |
| Lin 2021 | Multimorbidity | "coexistence of 2 or more of these 14 chronic diseases in the same individual" | Questions from CHARLS database | 14 | hypertension; dyslipidemia; diabetes; heart disease; stroke; chronic lung disease; asthma; liver disease; cancer; digestive disease; kidney disease; arthritis; psychiatric disease; memory-related disease (incl. Alzheimer's disease, Parkinson's disease, cerebral atrophy) | 58.9% |
| Mendizabal 2022 | Medical comorbidities + psychiatric comorbidities | NR | Clinical data taken from health records | NA | NA | NR |
| Noteboom 2021 | Chronic physical disorders | NR | Checklist of 17 physical disorders divided into 6 categories | 6 | cardiometabolic (hypertension, stroke, cardiac disease, diabetes); respiratory (asthma, COPD); musculoskeletal (hernia, chronic back disorders, osteoarthritis on knees, hands or hips, rheumatoid arthritis); digestive disorders (ulcers, serious bowel disorders); migraine; thyroid dysfunction | 7.2% |
| Patterson 2014 | Physical illness, mental disorders | NR | Author determined list of "30 chronic health conditions (lasting longer than six months)" | 30 | NR | NR |
| Post 2013 | Comorbidities | NR | Author determined list of conditions | 29 | allergies; arthritis; asthma; cancer; chronic fatigue syndrome; chronic menstrual irregularities; diabetes; fibromyalgia; head injury (with loss of consciousness); head injury (without loss of consciousness); heart disease; hypertension; hyperthyroidism; hypoglycaemia; hypotension; irritable bowel syndrome; kidney disease; liver disease/hepatitis; migraine headache; seizure; encephalitis; hyperadrenalism (Cushing's disease); hypoadrenalism (Addison's disease); meningitis; multiple sclerosis; narcolepsy; Parkinson's disease; stroke; other | NR |
| Reyes-Ortiz 2023 | Multimorbidity | "the coexistence of 2 or more chronic conditions" | Author determined list of conditions | 9 | hypertension; diabetes; coronary heart disease; arthritis; stroke; chronic pulmonary obstructive disease; osteoporosis; a mental (nervous, cognitive, or psychiatric) problem; cancer | 43.4%% |
| Sheikh 2018 | Morbidity | NR | Author determined list of "physical health outcomes". | 12 | heart attack; angina pectoris; stroke/brain haemorrhage; atrial fibrillation; osteoporosis; asthma; chronic bronchitis/emphysema/COPD; diabetes mellitus; hypothyroid/low metabolism; kidney disease (excluding urinary tract infection); migraine; hypertension | Mean 0.77; SD 0.99 |
| Sinnott 2015 | Multimorbidity | "the co-occurrence of two or more chronic diseases" | Author determined list of "common chronic diseases" | 20 | chronic back pain; hypertension; osteoarthritis; anxiety; rheumatoid arthritis; urinary incontinence; thyroid disease; asthma; depression; osteoporosis; other cardiac disease; diabetes; cancer; bronchitis; prior heart attack; angina, peripheral vascular disease; stroke; heart failure; aortic aneurysm | 45.3% |
| Sosnowski 2022 | Chronic physical health comorbidities | NR | Author determined list of conditions, "based on known associations with adverse childhood experiences…, prior comorbidities indices created in the ALIVE cohort, and medical conditions included in comorbidity burden indices". | 8 | diabetes; hypertension; cerebrovascular disease; renal disease; chronic lung disease; cancer; liver disease; obesity | 74.1% |
| Stapp 2020 | Medical morbidity | "concurrent medical conditions" | Author determined list of "past-year medical conditions" | 11 | arteriosclerosis; hypertension; cirrhosis; other liver disease; angina; tachycardia; myocardial infarction; other heart disease; stomach ulcer; gastritis; arthritis | 22.7% |
| Tomasdottir 2015 | Multimorbidity | "two or more coinciding chronic diseases or conditions" | Author determined list of "21 relevant disease conditions" | 21 | chronic back pain; obesity; hyperlipidaemia; mental health problems; hypertension; osteoarthrtis; asthma; cardiovascular disease; dental health problems; psoriasis; thyroidal diseases; gastro-esophageal reflux disease; cancer; fibromyalgia; diabetes; rheumatic arthritis; chronic obstructive pulmonary disease; renal diseases; osteoporosis; ankylosing spondylitis; epilepsy | 49.4% |
| Vasquez 2019 | Somatic multimorbidity + psychiatric multimorbidity | MM = "simultaneous presentation of two or more chronic diseases"  Somatic MM = "presence of two or more physical chronic conditions"  Psychiatric disorder MM = "presence of two or more DSM-5 12  months psychiatric diagnoses" | "list of prevalent and persistent conditions identified by the U.S. Department of Health and Human Services framework (USHHS) for multimorbidity measurement"  "a structured diagnostic interview, the NIAAA Alcohol Use Disorder and Associated Disabilities Interview Schedule-5 (AUDADIS-5)" | 22 | myocardial infarction; angina pectoris; tachycardia; other cardiovascular disease; hypertension; stroke; diabetes; arthritis; chronic bronchitis; emphysema; pneumonia; influenza; liver cancer; breast cancer; mouth/tongue/throat/oesophageal cancer; other cancer; mood disorders; anxiety disorders; post-traumatic stress disorder; substance use disorders; personality disorders; conduct disorders | NR |
| Yang 2021 | Multimorbidity | "the co-occurrence of two or more chronic diseases or medical conditions within the same individual" | Questions from CHARLS database | 14 | high blood pressure; diabetes; cancer; lung disease; heart problems; stroke; psychiatric problems; arthritis; dyslipidaemia; liver disease; kidney disease; digestive disease; asthma; memory problems | 40.2% |
| Zak-Hunter 2023 | Number of diseases | NR | Author determined list of conditions | 12 | type 1 diabetes; type 2 diabetes; heart disease; hypertension; food allergies; asthma; anorexia; bulimia; binge eating disorder; depression; gastrointestinal disorder; other | 4.9% |
| Zheng 2022 | Multimorbidity | "co-occurrence of two or more chronic non-communicable diseases" | Questions from CHARLS database | 14 | hypertension; dyslipidaemia; diabetes; cancer; chronic lung disease; liver disease; heart disease; stroke; kidney disease; digestive disease; asthma; arthritis; emotional, nervous or psychiatric problems; memory-related disease | NR |

NA = not applicable; NR = not reported; SD = standard deviation.

Figure S1: Meta-analysis of prevalence of exposure to ≥4 adverse childhood experiences

ACE = adverse childhood experience; CI = confidence interval.

Figure S2: Dose-response meta-analysis of the relationship between adverse childhood experiences and multimorbidity (using a non-linear/restricted cubic spline model)

ACEs = adverse childhood experiences.
